# Efficacy and Safety of Pancreatic Juice Cytology with Synthetic Secretin in Diagnosing Malignant Intraductal Papillary Mucinous Neoplasms of the Pancreas

**DOI:** 10.3390/diagnostics12030744

**Published:** 2022-03-18

**Authors:** Yohei Takeda, Kazuya Matsumoto, Takumi Onoyama, Taro Yamashita, Hiroki Koda, Wataru Hamamoto, Yuri Sakamoto, Takuya Shimosaka, Shiho Kawahara, Yasushi Horie, Hajime Isomoto

**Affiliations:** 1 Division of Gastroenterology and Nephrology, Department of Multidisciplinary Internal Medicine, Faculty of Medicine, Tottori University, Yonago 683-8504, Japan; golf4to@yahoo.co.jp (T.O.); yama_t11@yahoo.co.jp (T.Y.); po.polnga.3.negaiwo.xxx@gmail.com (H.K.); hamamoto_trr@yahoo.co.jp (W.H.); yuri.sakamoto@me.com (Y.S.); shimosaka.271@gmail.com (T.S.); kawahara.hp1@gmail.com (S.K.); isomoto@tottori-u.ac.jp (H.I.); 2 Division of Organ Pathology, Department of Pathology, Faculty of Medicine, Tottori University, Tottori 680-0000, Japan; mot7@me.com

**Keywords:** intraductal papillary mucinous tumor, pancreatic juice cytology, synthetic secretin

## Abstract

The risk of malignant transformation of intraductal papillary mucinous neoplasm (IPMN) is presently assessed using imaging, which remains unsatisfactory. Given the high viscosity of pancreatic juice, pancreatic juice cytology (PJC) is considered an investigational procedure. We previously demonstrated that the diagnostic performance of PJC was improved via synthetic secretin loading in pancreatic ductal carcinoma. This study aimed to evaluate the efficacy of synthetic secretin-loaded PJC (S-PJC) for IPMN. The usefulness and safety of S-PJC were prospectively evaluated in 133 patients with IPMN. Overall, 92, 12, and 26 patients had branch duct, main duct, and mixed-type lesions, respectively. The risk classifications based on the 2017 international consensus guidelines were high-risk stigmata, worrisome features, and no risk in 29, 59, and 45 patients, respectively. Synthetic secretin loading improved the sensitivity of PJC from 50.0% to 70.8%. Complications included 13 (9.8%) cases of mild pancreatitis, 1 (0.8%) case of acute cholangitis, and 1 (0.8%) case of Mallory–Weiss syndrome, all of which resolved with conservative treatment. In conclusion, synthetic secretin-loaded PJC improved the diagnostic performance of cytology for malignant IPMN. We recommend using synthetic secretin-loaded PJC for the preoperative pathological diagnosis of malignant IPMN in clinical settings.

## 1. Introduction

Benign or malignant intraductal papillary mucinous neoplasms (IPMNs) are mainly diagnosed based on imaging findings. Furthermore, the management of IPMN differs based on the diagnosis—the international consensus guidelines [1] recommend resection in cases classified as high-risk stigmata (HRS), while patients with worrisome features (WF) should be evaluated using endoscopic ultrasonography (EUS) to further stratify the lesion. Resection is recommended if any of the following are present: (1) definite mural nodule(s) ≥ 5 mm; (2) features suggestive of main duct (MD) involvement; and (3) cytology indicating or positive for malignancy. The rates of malignant IPMN in patients with HRS and WF are reported to be 59–65% and 30–35%, respectively, which are not sufficient [2,3]. EUS, fine-needle aspiration (FNA), and pancreatic juice cytology (PJC) in conjunction with endoscopic retrograde cholangiopancreatography (ERCP) are used for the pathological diagnosis of IPMN. One systematic review showed that EUS–FNA had a sensitivity of 57%, specificity of 84%, and an area under the curve of 0.82 for the diagnosis of malignant IPMN [4]. Moreover, the PIPE study showed that preoperative EUS–FNA for IPMN did not affect the frequency of peritoneal dissemination [5]. In contrast, in countries such as Japan, EUS–FNA is not performed for IPMN, owing to concerns of dissemination [6].

The sensitivity of PJC for malignant IPMN was found to be as low as 35% in a meta-analysis [7], and the accuracy was similarly unsatisfactory (33–51%) [2,8]. Furthermore, the incidence of post-ERCP pancreatitis (PEP) was reported to be approximately 8–25% [2,9,10]. Currently, several methods have been reported for improving the diagnostic ability of PJC, including brushing cytology, endoscopic nasal pancreatic juice drainage, cytodiagnosis with pancreatic duct lavage fluid, and PJC using synthetic secretin [11]. In 1992, Nakaizumi et al. reported the safety and high sensitivity of PJC using secretin for the diagnosis of pancreatic ductal adenocarcinoma (PDAC) in a porcine model [12]. We have previously reported that synthetic secretin increases the accuracy of PJC for PDAC [13], which is particularly useful in cases where EUS–FNA for PDAC is difficult to perform. The present study aimed to investigate improvements in the technique of using synthetic secretin for the diagnosis of malignant IPMN.

## 2. Methods

### 2.1. Study Design

In this single-center prospective study, a subgroup analysis of IPMN was conducted without controls. This study was approved by the Institutional Review Board of Tottori University Hospital in May 2011 (registration number: 2449). In 2019, the study protocol was partially upgraded, and it is now available in the University Hospital Medical Information Network Clinical Trials Registry (UMIN). The study was conducted in accordance with the Declaration of Helsinki for biomedical research involving human subjects. Information on all patients who underwent PJC has been prospectively entered into a database since May 2011.

### 2.2. Inclusion Criteria

The following inclusion criteria were used for patient selection: (1) no pathological diagnosis; (2) suspected malignant IPMN on imaging; and (3) age >20 years. All patients provided written informed consent for all procedures associated with the study. 

### 2.3. Eligibility Criteria for ERCP

**Figure 1:**
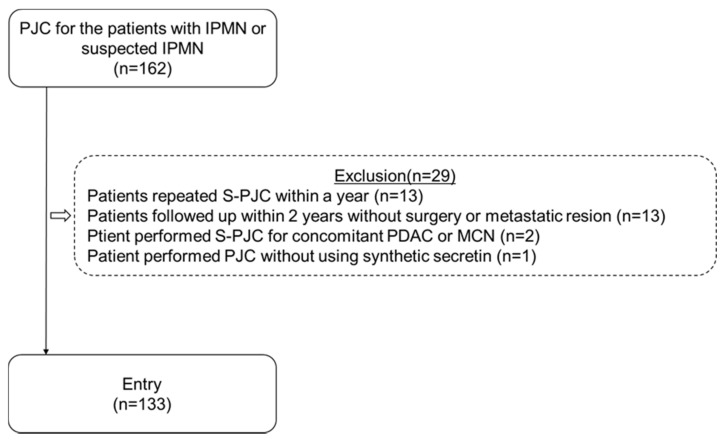
The algorithm used for patients’ inclusion and exclusion in this study. A total of 162 patients with IPMN or suspected IPMN were included. After excluding 29 patients (due to repeated S-PJC within a year, follow-up within 2 years without surgery or metastatic lesion, concomitant pancreatic ductal adenocarcinoma or mucinous cyst neoplasm, and the use of PJC without synthetic secretin), the remaining 133 patients were enrolled in this study. S-PJC: secretin-loaded pancreatic juice cytology; PJC: pancreatic juice cytology; PDAC: pancreatic ductal adenocarcinoma; MCN: mucinous cystic neoplasm; IPMN: intraductal papillary mucinous neoplasia.

We performed PJC in all patients enrolled in this study (Figure 1). In all cases, PJC was performed by endoscopists with at least 10 years of experience in performing ERCP. PJC was performed using a lateral-viewing endoscope (JF260 V, Olympus Optical Co., Ltd., Tokyo, Japan), a cannula (M, Boston Scientific Japan K.K., Tokyo, Japan; or PR−110 Q−1, Olympus Optical Co., Ltd., Tokyo, Japan; or Glo-Tip II^®^ Double Lumen ERCP Catheter, Cook Medical, Winston-Salem, North Carolina, USA), and a hydrophilic guidewire (M or M, Boston Scientific Japan K.K., Tokyo, Japan; or 228 ADBZX, ASAHI INTECC Co., Ltd., Aichi, Japan). No patient underwent endoscopic pancreatic stent placement.

### 2.4. Diagnosis and Classification of IPMN before ERCP

All patients underwent EUS along with computed tomography (CT) and/or magnetic resonance imaging (MRI), or both. A branch duct (BD)-IPMN was defined as a pancreatic cystic lesion with a diameter >5 mm that communicated with the MPD. An MD-IPMN was defined as segmental or diffuse dilatation of the MPD >5 mm without other causes of obstruction. Lesions that met the criteria for both BD-IPMN and MD-IPMN were categorized as mixed-type (MIX-IPMN) lesions. According to the revised international consensus guidelines for IPMN in 2017 [1], 29 patients were classified as having HRS and 59 as having WF. The remaining 45 patients were classified as having no risk (NR) in this study.

### 2.5. Synthetic Secretin and PJC

The synthetic secretin we used was ChiRhoStim (ChiRhoClin, Inc., Burtonsville, MD, USA) at the beginning of the study and Secrelux (Sanochemia, Vienna, Austria) after September 2013. In brief, the cannula was inserted into the MPD over the guidewire, and pancreatic juice was collected using a 10 mL syringe connected to the tip of the cannula in the MPD for 5 min. After collecting pancreatic juice, 0.6 μg of synthetic secretin was administered intravenously, and pancreatic juice was collected again for 5 min.

### 2.6. Cytodiagnosis

Papanicolaou staining was performed using conventional methods for PJC within 5 days of collecting pancreatic juice under ERCP. The aspirated material was evaluated by an expert cytopathologist (Y.H.). “Malignant” was defined upon noting the presence of malignant cells, irrespective of quantity. 

### 2.7. Follow-up

The final diagnosis was histologically verified using specimens obtained via surgical resection and/or EUS–FNA within 2 years, or based on the 2-year clinical course with CT imaging diagnosis or MRI diagnosis. For the resected specimens, low-to-intermediate-grade dysplasia was determined to be benign, and high-grade dysplasia and invasive carcinoma were determined to be malignant, according to the 2019 World Health Organization classification [14]. Patients without malignant disease were followed up with imaging examinations (CT, MRI, or EUS) every 6 months. All patients were closely observed for any immediate or delayed complications. PEP was diagnosed according to the diagnostic criteria reported by Cotton et al. [15].

### 2.8. Data Records

Data records included the location, size, Union for International Cancer Control (UICC) stage classification (eighth edition), endoscopic features of the lesion sampled, sample adequacy, cytology results, final diagnosis, and procedure-related complications. 

### 2.9. Endpoint

The primary endpoint was the sensitivity of S-PJC. We evaluated the sensitivity of PJC with and without using synthetic secretin. The secondary endpoints were the specificity and accuracy of S-PJC, rate of PEP, and quantity of pancreatic juice obtained from the MPD.

### 2.10. Statistical Analysis

The diagnostic power between subgroups was compared using the paired *t*-test, McNemar’s test, *t*-test, Fisher’s exact test, and Cochran–Armitage test. The significance level was set at *p* < 0.05. Various clinical factors associated with sensitivity were investigated on univariate and multivariate analyses. All statistical analyses were performed using EZR (Saitama Medical Center, Jichi Medical University, Saitama, Japan), which is a graphical user interface for R (The R Foundation for Statistical Computing, Vienna, Austria)—more precisely, it is a modified version of R Commander designed to add statistical functions frequently used in biostatistics [16].

## 3. Results

Overall, 13 patients were excluded because they underwent repeated S-PJC within 1 year, and we were concerned that retesting within 1 year might result in a false negative attributable to the influence of the initial test. Additionally, 13 patients were excluded due to follow-up within 2 years without death, surgery, or metastatic region by imaging diagnosis. Furthermore, two patients were excluded owing to S-PJC for concomitant PDAC or mucinous cyst neoplasm, whereas one patient was not included because PJC was performed without using synthetic secretin. Following the exclusion of these 29 patients, 133 patients were finally enrolled in the analysis. There were 83 men and 50 women, with age ranging from 50 to 87 years (mean: 71.3 years). Thirty patients underwent surgery after an examination. Table 1 shows the characteristics of patients with pancreatic disease.

Twenty-four patients were diagnosed with malignant IPMN within 2 years of follow-up after undergoing S-PJC. The mean time from S-PJC to the diagnosis of malignant IPMN was 141.6 ± 193.1 days (range: 4–721 days), <6 months in 20 cases, and between 6 months and 2 years in 4 cases. The rate of malignant IPMN was 62.1% (18/29) in patients with HRS, 10.2% (6/59) in patients with WF, and 0% (0/45) in patients with NR. Table 2 shows the frequencies of malignant IPMN for HRS and WF. 

The rate of malignant IPMN was 69.6% (16/23) in enhancing mural nodules > 5 mm, which accounted for most cases of HRS (Table 3).

**Figure 2:**
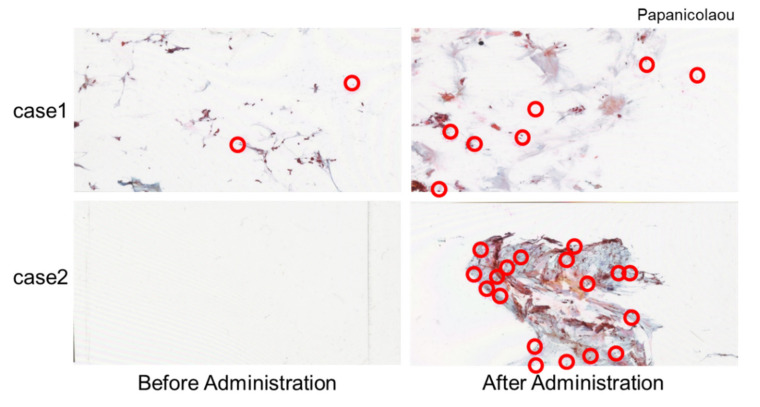
Comparison of cell numbers in the pancreatic juice before and after synthetic secretin administration. After synthetic secretin administration, cell clumps (red circles) were increased in both cases 1 and 2.

The average quantity of pancreatic juice obtained was increased from 3.7 ± 7.3 mL (range: 0–79.0 mL) to 5.1 ± 6.2 mL (range: 1–64.0 mL) after synthetic secretin administration (*p* < 0.001) (Table 4). In some cases, there were more cell clumps after synthetic secretin administration, as compared to before loading (Figure 2). Pancreatic juice was collected by S-PJC in 131 cases (98.5%). We could not insert the catheter into the main pancreatic duct (MPD) through the papilla in two cases.

Table 5 summarizes the diagnostic power of S-PJC. The sensitivity, specificity, and accuracy of PJC prior to synthetic secretin administration were 50.0%, 91.7%, and 84.2%, respectively. When all PJC measurements were combined, the results were significantly increased to 70.8%, 90.8%, and 87.2%, respectively (sensitivity and accuracy: before administration vs. total, *p* = 0.031). 

Among 133 patients who underwent ERCP for S-PJC, 13 (9.8%) developed PEP, whereas one (0.8%) developed cholangitis and Mallory–Weiss syndrome. PEP complication rates exhibited no significant association with sex, age, malignancy, risk classification, or macroscopic type. All cases of PEP were mild, and all patients were cured with conservative medical treatment. There were no adverse events thought to be due to synthetic secretin.

## 4. Discussion

As per the international consensus guidelines for IPMN revised in 2017 [1], HRS is considered an indication for resection, while WF warrants further investigations, which include EUS and PJC. EUS findings are defined as (1) definite mural nodules ≥5 mm and (2) features suspicious for MD involvement. The rates of malignant IPMN in patients with HRS and WF have been reported to be 59–65% and 30–35%, respectively [2,3]. The diagnostic ability of imaging studies for malignant IPMN is limited. A clinical study following the flowchart of international consensus guidelines reported that PJC improved the sensitivity of WF [2].

Nevertheless, the use of PJC for diagnosing benign and malignant IPMN is problematic because of its low sensitivity. The results of a meta-analysis revealed a sensitivity of 35.1%, specificity of 97.2%, and positive diagnosis rate of 92.9%, indicating that washing cytology improved sensitivity [7]. KL−6 measurement [17], immunostaining for factors such as Ki−67, p53, and MUC using a cell block method [10], and liquid-based cytology for pathological specimen treatment have been shown to improve the sensitivity [3]; however, despite the use of these methods, the diagnostic ability of PJC for malignant IPMN remains insufficient. 

Previously, PDAC was frequently diagnosed with PJC performed using porcine secretin, the usefulness of which has been reported previously [12]. However, due to the risk of infection, porcine secretin is no longer used in most countries. In 2004, the US FDA approved human synthetic secretin, thereby bringing it back into focus. The FDA has approved human synthetic secretin for (!) the stimulation of pancreatic secretions containing bicarbonate, (2) aiding the diagnosis of pancreatic exocrine dysfunction, (3) the use of gastrin secretions to assist in the diagnosis of gastrinoma, and (4) the stimulation of pancreatic secretions to help identify the ampulla of Vater and accessory papilla during ERCP. There are only a few reports on the administration of human synthetic secretin in the diagnosis of malignant IPMN, such as studies using digital next-generation sequencing and magnetic resonance cholangiopancreatography (MRCP) [18,19]. Although there are reports of PJC performed using secretin, researchers did not compare the diagnostic performance of PJC with and without secretin [20].

This is the first report on the utility and safety of using synthetic secretin for the pathological diagnosis of malignant IPMN. Synthetic secretin loading improved the sensitivity of PJC from 50.0% to 70.8%. In terms of complications, there were 13 cases of mild pancreatitis (9.8%), 1 case of acute cholangitis (0.8%), and 1 case of Mallory–Weiss syndrome (0.8%); all patients improved with conservative treatment. Meanwhile, HRS had a positive predictive value of 62.1% (18/29), and WF had a positive predictive value of 10.2% (6/59).

Of the cases with WF, 10 were malignant at the time of S-PJC, and 5 developed malignant IPMN at follow-up within 2 years of S-PJC. Among the remaining five cases, one case was diagnosed as a malignant IPMN at 32 months after S-PJC, whereas one case was diagnosed at 45 months after S-PJC. The process of carcinogenesis in IPMN is relatively slow; nevertheless, once atypia occurs, it may rapidly proceed to carcinogenesis [21,22]. Of the cases included in this study, five were diagnosed with malignant IPMN more than two years after S-PJC, the intervals being 817, 967, 1358, 2025, and 2976 days from S-PJC. Among these, two cases were diagnosed as malignant on S-PJC, and three were diagnosed as benign.

In terms of treatment, international consensus documents recommend the resection of IPMN when a malignancy is suspected. On the other hand, because of the advanced age of many patients, other unpredictable complications are likely to be prognostic factors, and the length of time before the intraepithelial carcinoma is defined as invasive intraductal papillary mucinous carcinoma needs to be considered in terms of whether resection of the intraepithelial carcinoma necessarily contributes to the prognosis. The IPMN International Practice Guidelines were first published in 2006 [23], and revised in 2012 [24] and 2017 [1]. With each edition, the imaging findings suspicious for malignant IPMN have changed. In this study, all diagnoses were made based on the 2017 edition.

The present study has some limitations. (1) Given the complexity of the procedure, the endoscopist performing the PJC should ideally have at least 10 years of ERCP experience, and facilities performing S-PJC should be limited to referral centers. (2) The present study was limited to a single center. (3) A single pathologist (Y.H.) evaluated the pathological findings. (4) Adequate medical and technical preventive assessment procedures for PEP were not performed. (5) Finally, synthetic secretin has not been approved for use in Japan by the Pharmaceutical Affairs Law in Japan, and we imported it after obtaining approval from the ethics review committee of our hospital. Therefore, availability may be a key challenge for other researchers wishing to conduct similar studies in Japan.

In conclusion, synthetic secretin administration increased the amount of pancreatic juice collected and improved the diagnostic performance of cytology. Synthetic secretin-loaded PJC can be useful for the preoperative pathological diagnosis of malignant IPMN in clinical settings.

## Figures and Tables

**Table 1:** Characteristics of patients with pancreatic disease.

Factors	
Sex, M/F	83/50
Age, years, range (mean)	50–87 (71.3)
IPMN (surgical cases), *n*	109 (5)
Malignant IPMN (surgical cases), *n*	24 (21)
Size (BD-IPMN), mm	4–94 (26.6)
Macroscopic type, *n*	
BD	95
MIX	26
MD	12
Risk classification according to the 2017 ICG, *n*	
HRS	29
WF	59
NR	45
Stage ^†^ (0/IA/IB/IIA/IIB/III/IV), *n*	10/5/2/2/2/1/2

^†^ Union for International Cancer Control staging system for pancreatic ductal adenocarcinoma (PDAC). BD: branch duct; IPMN: intraductal papillary mucinous neoplasm; MIX: mixed-type; MD: main duct; ICG: international consensus guidelines; HRS: high-risk stigmata; WF: worrisome features; NR: no risk.

**Table 2:** Final diagnostic results for each risk classification according to the 2017 ICG.

		Final Diagnosis		
		IPMN (*n*)	Malignant IPMN (*n*)	Total (*n*)
Risk classification according to the 2017 ICG	HRS	11	18	29
	WF	53	6	59
	NR	45	0	45

IPMN: intraductal papillary mucinous neoplasm; HRS: high-risk stigmata; WF: worrisome features; NR: no risk; ICG: international consensus guidelines.

**Table 3:** Rate of malignant IPMN for each imaging finding.

Factors	(%)	*n*
Obstructive jaundice	100%	1/1
Enhancing mural nodule ≥ 5 mm	69.6%	16/23
Main pancreatic duct ≥ 10 mm	61.5%	8/13
Past history of pancreatitis	N.A.	0/0
Cyst ≥ 3 cm	29.2%	14/48
Thickened/enhancing cyst walls	25.0%	5/20
Main pancreatic duct size of 5–9 mm	37.5%	12/32
Abrupt change in caliber of pancreatic duct with distal pancreatic atrophy	66.7%	2/3
Lymphadenopathy	100%	2/2
Cyst growth rate over ≥ 5 mm/2 years	0%	0/1

IPMN: intraductal papillary mucinous neoplasm; N.A.: not applicable.

**Table 4:** Comparison of pancreatic juice quantity before and after synthetic secretin administration.

Synthetic Secretin	Amount of Pancreatic Juice	*p*-Value
Before administration	3.7 ± 7.3 mL (0–79.0 mL)	<0.001 ^†^
After administration	5.1 ± 6.2 mL (1–64.0 mL)	

^†^ Paired *t*-test.

**Table 5:** Diagnostic ability of pancreatic juice cytology.

Synthetic Secretin	Adequate Specimen	Sensitivity	Specificity	PPV	NPV	Accuracy
Before administration	98.5% (131/133)	50.0% (12/24 *)	91.7% (100/109)	57.1% (12/21)	89.3% (100/112)	84.2% (112/133)
After administration	98.5% (131/133)	54.2% (13/24)	93.6% (102/109)	65% (13/20)	90.3% (102/113)	86.5% (115/133)
Total	98.5% (131/133)	70.8% (17/24 *)	90.8% (99/109)	63.0% (17/27)	93.4% (99/106)	87.2% (116/133)

* Statistically significant when compared to the total value by McNemar’s test (*p* = 0.031). NPV: negative predictive value; PPV: positive predictive value.

## Data Availability

The datasets generated and/or analyzed during the current study are available from the corresponding author upon reasonable request.
